# Interactions between herbivory and warming in aboveground biomass production of arctic vegetation

**DOI:** 10.1186/1472-6785-8-17

**Published:** 2008-10-22

**Authors:** Christian Pedersen, Eric Post

**Affiliations:** 1 Department of Biology, Penn State University, 208 Mueller Lab, University Park, PA 16802 USA; 2 World Wildlife Fund Norway, P.O. Box 6784 St. Olavs Plass, 0130 Oslo, Norway; 3 Department of Arctic Environment, NERI-University of Aarhus, Frederiksborgvej 399, DK-4000 Roskilde, Denmark

## Abstract

**Background:**

Many studies investigating the ecosystem effects of global climate change have focused on arctic ecosystems because the Arctic is expected to undergo the earliest and most pronounced changes in response to increasing global temperatures, and arctic ecosystems are considerably limited by low temperatures and permafrost. In these nutrient limited systems, a warmer climate is expected to increase plant biomass production, primarily through increases in shrubs over graminoids and forbs. But, the influence of vertebrate and invertebrate herbivores has been largely absent in studies investigating the effects of vegetation responses to climate change, despite the fact that herbivory can have a major influence on plant community composition, biomass and nutrient cycling. Here, we present results from a multi-annual field experiment investigating the effects of vertebrate herbivory on plant biomass response to simulated climate warming in arctic Greenland.

**Results:**

The results after four years of treatments did not give any clear evidence of increased biomass of shrubs in response climate warming. Nor did our study indicate that vertebrate grazing mediated any increased domination of shrubs over other functional plant groups in response to warming. However, our results indicate an important role of insect outbreaks on aboveground biomass. Intense caterpillar foraging from a two-year outbreak of the moth *Eurois occulta *during two growing seasons may have concealed any treatment effects. However, there was some evidence suggesting that vertebrate herbivores constrain the biomass production of shrubs over graminoids and forbs.

**Conclusion:**

Although inconclusive, our results were likely constrained by the overwhelming influence of an unexpected caterpillar outbreak on aboveground biomass. It is likely that the role of large vertebrate herbivores in vegetation response to warming will become more evident as this experiment proceeds and the plant community recovers from the caterpillar outbreak. Due to the greater influence of invertebrate herbivory in this study, it is advisable to consider both the effect of invertebrate and vertebrate herbivores in studies investigating climate change effects on plant communities.

## Background

Arctic ecosystems have been a major focus of climate change studies because biological processes in the northern high-latitude environments are considerably limited by temperature and the existence of permafrost. Records show that mean winter temperatures over northern continents have increased considerably in the last 30–40 years [1] while paleoclimate evidence (e.g. sediments, tree rings and glaciers) suggest that the Arctic has now warmed to the highest temperatures in the last 400 years [2]. Arctic ecosystems are generally nutrient (nitrogen and phosphorus) limited, and climatic warming is expected to increase nutrient mineralization and decomposition rates [3]. This increase in availability and turnover of limited nutrients is predicted to have a positive influence on net primary production (NPP) and cause an increase in plant biomass of arctic vegetation [3]. Process models indicate that the NPP response of arctic vegetation to increasing CO_2 _concentration in the atmosphere is dependent on increased nitrogen mineralization due to climate warming [4]. Increased NPP might in turn compensate for carbon loss from arctic soils by sequestering carbon as increased plant biomass production resulting from increased availability of mineralized nitrogen [5]. In Alaskan tundra, for instance, annual aboveground plant production doubled in response to experimental fertilization [6].

Productivity responses at the community level are dependent on species diversity, plant community composition, and plant growth forms (functional groups). Higher species diversity is often correlated with higher primary production and biomass accumulation [7]. Several studies have reported differential effects of climate change on biomass production of plant functional groups and, hence, plant community composition. In arctic Alaska, species diversity declined following warming due to the increasing dominance of shrubs at the expense of graminoids and forbs [8,9]. This might again affect the carbon balance in the Arctic because the carbon storage potential of shrubs is higher than that of graminoids and forbs [10,11]. Recent observations of increased shrub abundance in arctic Alaska [11], as well as a meta-analysis of warming experiments at 13 arctic research sites, showed increasing dominance of shrubs over other functional plant groups [12], and suggests that there is a potential for increased ecosystem carbon storage in the Arctic in response to future warming (but see Mack et al. 2004).

However, herbivory may be an important and overlooked component of primary productivity responses to warming and plant community dynamics. Experiments addressing the effect of grazing or browsing in several ecosystems have revealed that vertebrate herbivores have a considerable impact on plant biomass, NPP, nutrient cycling and species composition [13-20]. These influences may mediate the response of vegetation to climate change, and may be especially important in the Arctic, where productivity is generally nutrient limited [21,22]. In Greenland, grazing by caribou has been reported to cause die-backs of dwarf birch (*Betula nana*) and the spread of *Poa *sp. dominated grazing lawns [23]. Both studies of caribou summer ranges in sub-Arctic Canada [17] and 30 year old herding fences on reindeer pastures in Northern Norway [24] suggest that grazing and trampling both have the potential to constrain productivity and reduce standing biomass of shrubs in the Arctic. Vertebrate herbivores have also been shown to reduce the depth of the moss layer through grazing and trampling, which could further affect soil temperatures and nutrient cycling [24-27]. Studies of 40-year old reindeer herding fences in northern Norway have revealed that reindeer grazing and trampling has produced a shift in tundra vegetation from moss-rich to graminoid-dominated meadows [24], and resulted in increased soil temperatures, decomposition rates and nutrient availability [25]. Similar results have been found in exclosure experiments on Svalbard, where reductions in the moss layer have resulted in higher soil temperatures, increased litter decomposition, and enhanced primary production [26]. The overall effect of vertebrate herbivory will depend on the balance between negative and positive feedback effects that such herbivores exert on plant productivity and nutrient cycling [15]. Neglecting the influence of vertebrate herbivores on plant productivity may bias estimates of ecosystem changes in relation to global warming in areas where large vertebrate herbivores occur.

Here, we present results from a multi-annual field experiment conducted on the summer range of the Kangerlussuaq-Sisimiut caribou herd in West-Greenland. Using herbivore exclosures and open-top chambers (OTCs), we investigated the influence of caribou and muskox grazing on the aboveground biomass response of arctic vegetation to warming. We hypothesized that herbivory by caribou and muskoxen has the potential to suppress growth of shrubs and promote development of graminoid-dominated swards, thereby mediating the expected accumulation of biomass in response to warming where these herbivores occur.

## Results and discussion

### Temperature treatments

**Figure 1:**
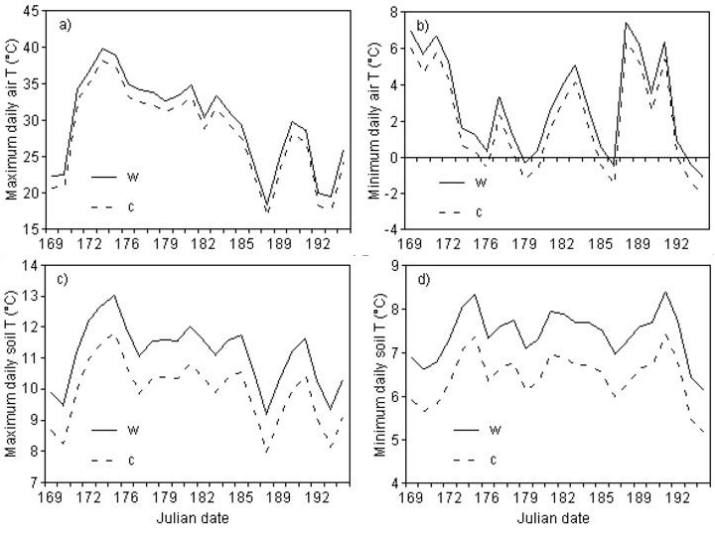
**Mean daily temperatures for warmed (w) and ambient (control, c) plots measured during the warming treatments.** (a) Maximum near surface air temperature was on average 29.7°C in the warmed and 28.1°C in the control plots. (b) Minimum near surface temperature was on average 2.9°C in the warmed and 2.0°C in the control plots. (c) Maximum soil temperature at 10 cm depth was on average 11.1°C in the warmed plots and 9.9°C in the control plots. (d) Minimum soil temperature at 10 cm depth was on average 7.4°C in the warmed plots and 6.5°C in the control plots.

Maximum daily near surface temperatures (Figure 1a) were on average 1.6°C higher on warmed than on control plots (F = 9.7, p = 0.002) for the entire period of measurements. Minimum daily near surface temperatures (Figure. 1b) were on average 1.0°C higher on warmed than on control plots (F = 47.4, p < 0.001). Maximum daily soil temperatures, measured at 10 cm depth, were on average 1.2°C higher on the warmed plots than on the control plots (F = 45.7, p < 0.001) (Figure. 1c), while minimum daily soil temperatures (Figure. 1d) were on average 1.0°C higher on the warmed plots than on the control plots (F = 28.5, p < 0.001).

The OTC treatment did not increase soil or near surface temperatures as much as reported from similar experiments elsewhere, where a warming effect of 3–4°C has been reported [28-31]. Nor was the temperature increase measured in this study within the range of an additional arctic warming of 4–7°C predicted by the ACIA for the next 100 years [32]. The maximum and minimum daily near surface temperatures were on average only 1.6°C and 1.0°C higher in the OTC's compared to the controls, respectively. Maximum and minimum daily soil temperatures differed by only 1.2°C and 1.0°C on average, respectively. It is therefore questionable if these relatively small differences in temperature could induce a change in plant production similar to what is expected during predicted climate warming. Several other studies [3,8,33,34] have shown that increased nutrient availability has the strongest effect on plant production, and that temperature has an indirect effect through increased nutrient mineralization rates in the soil. The relatively small increase in soil temperature observed in this study might not have been enough to stimulate mineralization rates in the plots comparable to nutrient concentrations added in other experiments. On the other hand, the warming effect achieved in this study was closer to the increase of 0.2°C per decade in global average temperature observed during the last three decades [35], and might therefore have been a more realistic warming treatment compared to what is expected to occur during a short term experiment.

### Above ground biomass

**Figure 2:**
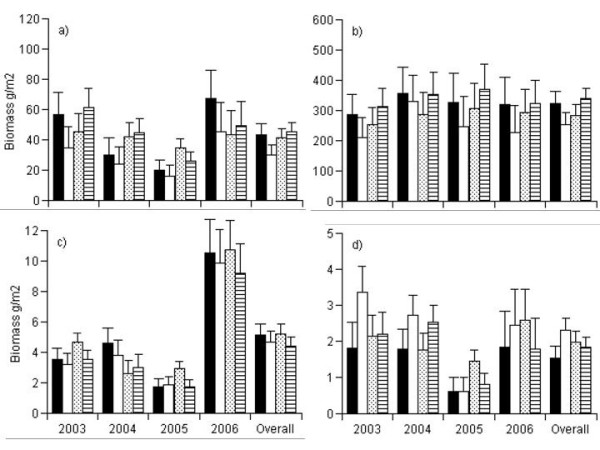
**Mean biomass (g/m^2^) of major plant functional groups for each of the treatment combinations during the four years of warming treatments in low shrub tundra, Kangerlussuaq, Greenland.** Biomass was estimated by calibration of the non-destructive point-intercept method with actual plant biomass measurements. a) *B. nana *leaves, b) *B. nana *stem, c) Graminoids and d) Forbs. Black columns: ungrazed and ambient; white columns: ungrazed and warmed; light gray columns: grazed and ambient; and dark gray columns: grazed and warmed. Error bars are the SE of the means.

When aboveground biomass was analyzed across all years, "year" was highly significant for all plant functional groups (Table 1), and there was a significant interaction between the exclosure and warming treatments for *B. nana *stems. There was a strong reduction in biomass from 2003 until 2005, before there was a marked increase in biomass in 2006 (Figure 2). This change appeared to some extent to depend on the treatments, but the overall strong reduction in aboveground biomass was due to the moth outbreak in 2004 and 2005, causing a significant "year" effect (Figure 2), and potentially swamping out the warming and exclosure treatments.

**Table 1:** Results from the nested analyses of variance of treatment effects on the biomass (g/m^2^) of plant functional groups in low shrub tundra, Kangerlussuaq, Greenland

	Source of variation									
	
Variable	W		E		Y		E*W		E*W*Y	
	F	p	F	p	F	p	F	p	F	p
	
*B. nana *leaf	1.051	0.332	1.677	0.221	10.546	0.003^a^	3.365	0.099	0.627	0.771
*B. nana *stem	0.330	0.579	2.460	0.130	8.780	0.004^a^	24.907	0.001^a^	0.122	0.999
Graminoids	4.363	0.065	0.074	0.789	100.602	<0.001^a^	0.517	0.490	0.228	0.990
Forbs	1.777	0.215	0.000	0.985	10.216	0.003^a^	3.730	0.085	0.646	0.754

W: warming treatment, E: exclosure treatment, Y: year. ^a ^marks significance level of p ≤ 0.05.

For *B. nana *leaves, on the plots exclosed from grazing, biomass was significantly higher in 2003 and 2006 than in 2004 and 2005 (Figure 2a). However, on the plots exposed to grazing there appeared to be no significant differences between the years. For *B. nana *stems there were no significant differences between any of the years (Figure 2b).

For graminoids there appeared to be the same pattern for all treatment combinations with biomass measured in 2006 significantly higher than in all previous years (Figure 2c). The biomass of forbs was significantly lower in 2005, the peak of the caterpillar outbreak, than in any of the other years for all the treatment combinations except for the grazed-ambient (GA) plots (Figure 2d). For the GA plots, the biomass observed in 2005 was only significantly lower than that observed in 2006.

When the different temporal responses in biomass were analyzed, few significant effects of the treatments were noted (Table 2). There was a marginally significant secondary (δ_p_) effect of the exclosure treatment for *B. nana *leaves, while graminoids showed a significant primary (δ_p_) response to the exclosure treatment (Table 2) (see Methods for definitions of the terms "secondary" and "primary" in relation to plant biomass dynamics).

**Table 2:** Results from the nested analyses of variance of treatment effects on the changes in biomass (g/m^2^) of major plant functional groups in low shrub tundra, Kangerlussuaq, Greenland

		P-values		
		
Variable	Response	W	E	E*W
B. nana leaf	δ_p_	0.862	0.291	0.097
	δ_s_	0.898	0.056	0.225
	δ_c_	0.751	0.191	0.716
B. nana stem	δ_p_	0.512	0.093	0.594
	δ_s_	0.698	0.887	0.895
	δ_c_	0.609	0.965	0.864
Graminoids	δ_p_	0.474	0.021^a^	0.188
	δ_s_	0.696	0.995	0.960
	δ_c_	0.802	0.916	0.993
Forbs	δ_p_	0.891	0.484	0.293
	δ_s_	0.738	0.509	0.725
	δ_c_	0.297	0.736	0.875

δ_p_: primary change 2003–2004, δ_s_: secondary change 2005–2006, δ_c_: cumulative change 2003–2006. W: warming treatment, E: exclosure treatment. ^a ^indicates significance at the p ≤ 0.05 level.

During the primary response (δ_p_), the only noticeable effect was a significant increase in *B. nana *stem biomass for all treatment combinations (Table 3). For the secondary response (δ_s_), there were significant positive changes in aboveground biomass for all the treatment combinations, except for *B. nana *stems (Table 3). There were significant and positive cumulative responses (δ_c_) in aboveground biomass for *B. nana *stem, graminoids and *B. nana *leaves on the ungrazed plots (Table 3).

**Table 3:** Mean changes in biomass (g/m^2^) of major plant functional groups in low shrub tundra, Kangerlussuaq, Greenland

		δ_p_		δ_s_		δ_c_	
		
Functional groups	Treatment combinations	Mean change (g/m^2^)	95% CI [min, max]	Mean change (g/m^2^)	95% CI [min, max]	Mean change (g/m^2^)	95% CI [min, max]
B. nana leaf	Ungrazed Ambient	-10.03	[-28.86, 8.80]	74.85^a^	[46.32, 103.38]	35.03^a^	[2.14, 67.91]
	
	Ungrazed Warmed	6.69	[-12.14, 25.52]	56.61^a^	[28.08, 85.13]	35.75^a^	[2.87, 68.64]
	
	Grazed Ambient	14.63	[-2.86, 32.11]	31.55^a^	[5.06, 58.03]	20.02	[-10.51, 50.55]
	
	Grazed Warmed	0.96	[-16.50, 18.47]	46.37^a^	[19.88, 72.86]	9.49	[-21.04, 40.02]

B. nana stem	Ungrazed Ambient	214.67^a^	[124.71, 304.54]	103.41	[-34.61, 241.42]	159.29^a^	[55.84, 262.75]
	
	Ungrazed Warmed	264.98^a^	[175.07, 354.89]	86.81	[-51.21, 224.82]	142.69^a^	[39.24, 246.15]
	
	Grazed Ambient	163.75^a^	[80.28, 274.22]	102.722	[-25.41, 230.85]	165.45^a^	[69.41, 261.50]
	
	Grazed Warmed	168.97^a^	[85.49, 252.44]	69.05	[-59.08, 197.18]	132.25^a^	[36.21, 228.30]

Graminoids	Ungrazed Ambient	0.83	[-1.02, 2.69]	8.04^a^	[4.20, 11.89]	6.46^a^	[2.40, 10.53]
	
	Ungrazed Warmed	0.29	[-1.56, 2.14]	7.25^a^	[3.40, 11.09]	6.00^a^	[1.93, 10.07]
	
	Grazed Ambient	-2.48^a^	[-4.20, -0.76)]	7.94^a^	[4.37, 11.51]	6.28^a^	[2.50, 10.05]
	
	Grazed Warmed	-0.69	[-2.41, 1.03]	7.33^a^	[3.76, 10.89]	5.78^a^	[2.01, 9.56]

Forbs	Ungrazed Ambient	-0.51	[-2.66, 1.64]	2.17^a^	[0.19, 4.14]	0.67	[-1.67, 3.00]
	
	Ungrazed Warmed	-1.72	[-3.87, 0.43]	2.80^a^	[0.83, 4.77]	-0.66	[-2.99, 1.67]
	
	Grazed Ambient	-0.88	[-2.87, 1.12]	1.88^a^	[0.04, 3.71]	0.86	[-1.30, 3.03]
	
	Grazed Warmed	0.06	[-1.93, 2.06]	1.86^a^	[0.03, 3.69]	-0.12	[-2.28, 2.05]

CI: confidence intervals of the means for each treatment combination. ^a ^indicates that the mean is statistically different from 0 at p ≤ 0.05. δ_p_: primary change (2003–2004), δ_s_: secondary change (2005–2006), δ_c_: cumulative change (2003–2006).

The purpose of this experiment was to investigate the joint effects of ungulate herbivores and warming on the aboveground biomass response of key plant functional groups in a low shrub tundra ecosystem. However, we acknowledge that the warming manipulations did not reflect all the possible environmental changes that are likely to occur as a consequence of increased greenhouse gas concentrations in the atmosphere, including increased precipitation, changes in precipitation and timing of snowmelt, and increasing length of the growing season. However, OTCs were in place most years before snow melt, and likely influenced timing of snowmelt and initiation of the growth season within the warmed plots. Our intention was to focus on one climatic factor that is predicted to have both direct and indirect influences on aboveground biomass of arctic plants. Moreover, it is throughout the plant growth season that vascular plants are most exposed to ungulate grazing. Finally, it should be emphasized that our OTC treatments are comparable to those of other experimental studies of climate change in the Arctic, and will facilitate comparisons of results, with the added dimension of herbivory.

Our results do not provide any clear indication that the warming and exclosure treatments alone or in concert had any effect on inter-annual dynamics of above-ground biomass production by any of the plant functional groups during the first four years of warming and exclosure treatments. No significant differences in shrub (*B. nana*), graminoid or forb biomass were found for any of the treatment combinations in any of the years. This result is somewhat surprising since several other studies have found clear indications of increasing shrub biomass and abundance at the expense of non-vascular plants to experimental warming [12,36]. However, the majority of these experiments have found that it is the addition of nutrients alone or in combination with warming that produces the strongest responses [8-10,37]. Furthermore, the unexpected moth outbreak during 2004 and 2005 made a strong impact on aboveground biomass of all the plant functional groups and also potentially influenced nutrient dynamics. This highly pulsed event makes it difficult to draw conclusions about the experimental treatment effects, but some weak responses were found. Our data from 2006 suggest that the biomass of *B. nana *leaves and graminoids had begun to recover from the moth outbreak in that year (Figure 2), suggesting that additional years of warming and exclosure may be needed to detect effects of those treatments.

To some extent, the lack of strong responses found in this study lends support to the hypothesis that it is the indirect effects of cold temperatures that limit plant production through low nutrient availability, rather than low temperatures per se [37]. It has been shown that nutrient addition elicits a stronger response on plant biomass than increased temperature [38]. Long-term nutrient addition and temperature manipulations in arctic ecosystems have revealed that elevated temperature can indirectly affect plant biomass and productivity by increasing nitrogen and phosphorous availability through increased soil mineralization rates [8], while nutrient addition directly increases biomass production and alters plant community composition [8,39].

There might be other explanations for the limited response in biomass production as well. The largest short-term effects of warming might not appear as responses in biomass but rather in other important plant traits such as flower and seed production [10,36]. Furthermore, studies that found effects of warming treatments on biomass involved temperature manipulations of 2–3°C higher than what was achieved in this study. Such a temperature difference could facilitate a much stronger biomass response.

The exclosure treatment was expected to increase aboveground biomass on the ungrazed plots compared to the plots exposed to grazing, especially for shrubs [16,17,23,24]. Furthermore, the effect of grazing was expected to promote the production of graminoids and forbs. However, we found no detectable effects of the exclosure treatment at this stage of the experiment, possibly because this treatment was negated by the moth outbreak. The strongest effect we detected was that of "year". There was a reduction in aboveground biomass from 2003 until 2005 for all of the functional groups, followed by a strong increase in biomass from 2005 to 2006. Only the stem biomass of *B. nana *did not show the same strong reduction from 2003 to 2005. This corresponds with the outbreak of the moth *E. occulta *that first reached noticeable numbers in 2004 and reached even higher numbers in 2005. The strong reduction in leaf biomass, together with the lack of an effect on stem biomass, suggests that the shrub *B. nana *was more affected by caterpillar foraging than by our manipulations. It is quite possible that the effect of the exclosures and OTCs on biomass production may have been concealed by the biomass off-take of caterpillar foraging. The density of caterpillars during the moth outbreak was found not to interact with the warming treatment, indicating that pulse herbivory by caterpillars reduced the plant community biomass response to warming [40].

Although our distinction between primary and secondary biomass responses is arbitrary due to the short duration of the experiment, these indications can still be useful in understanding any effects on inter-annual biomass production, especially when it comes to the direction and magnitude of change. There were very few significant temporal effects found in response to our treatments. But there was a significant increase in stem biomass for *B. nana *for all the treatment combinations for the primary and cumulative responses (Table 3). This change in biomass is larger in the plots located inside the exclosures and suggests that grazing has a limiting effect on biomass accumulation in shrubs in response to climate warming.

Furthermore, during the secondary response, there were significant positive changes in leaf biomass of *B. nana *for all treatment combinations; however none of our manipulations produced any changes in stem biomass. This difference in response between leaf- and stem biomass could indicate a resource allocation strategy as a response to the relatively heavy defoliation these plants experienced during the 2004 and 2005 moth outbreak. Plants have been shown to exhibit such a compensational strategy in growth as a response to herbivory [41-45], and what we have observed here could be a response to the limited photosynthetic capacity they experienced during the caterpillar outbreak that removed nearly all the leaf biomass [40].

For both graminoids and forbs, the secondary change in biomass was significant and positive for all treatment combinations. This is most likely a recovery from the caterpillar outbreak, but might also be a response to the limited increase in *B. nana *stem biomass. When the cumulative change was examined, the change in leaf biomass of *B.nana *was only significant on the ungrazed plots, suggesting a negative effect of grazing. For graminoids, there was a significant increase in biomass, but no difference between the treatment combinations, suggesting a limited response to our manipulations.

Overall, there was substantial variation in the direction and magnitude of the different temporal responses in this experiment. The findings lend support to other studies that have found that short-term responses of vegetation to warming are not good predictors of longer term trends [8,28,33].

To reach a general conclusion about the variation in direction and magnitude of the changes in biomass among functional groups observed in this study is difficult and complex. Interpretation of these results is further complicated by the outbreak of caterpillars of the moth *E. occulta*, which most likely is the main reason why several of the plots showed a reversal from a neutral to a sometimes strong positive change in biomass. Our results indicate, thereby, an important role of highly pulsed herbivory, such as the caterpillar outbreak, on aboveground biomass. What remains unaddressed in this study is how both invertebrate and vertebrate herbivory will influence long-term changes in this ecosystem. Such a general conclusion can only be reached as this experiment progresses.

## Conclusion

The small observed responses to the warming and exclosure treatments presented here could be an indication that our study area is predominantly nutrient limited, and that nutrient limitation may explain the lack of responses during the initial stages of this long term experiment. On the other hand, if this system is not nutrient limited, then increased availability of nutrients due to climate warming might not result in increased biomass production. Our preliminary results provide only limited evidence that vertebrate herbivory constrains the biomass response of shrubs to warming, and that grazing promotes establishment of graminoids and forbs. The overall strongest effect was from the unexpected moth outbreak. This highly pulsed event caused a reduction in aboveground biomass that likely concealed any treatment effect. Although modest, there were changes in the biomass of the plant functional groups studied here, suggesting that vertebrate herbivory could mediate the competitive dominance of shrubs over graminoids that is a predicted response to climate change. Furthermore, these results also indicate a limited response in forb biomass which could indicate that species of this functional group are more vulnerable to warming and herbivory than the other functional groups. The differences in the short-term and long-term responses also suggest that the short duration of this study may prevent general conclusions from being made about the interacting effects of climatic warming and herbivory. However, as this experiment progresses, more of the mechanisms controlling biomass responses of the vegetation should be revealed.

## Methods

### Study area

The study was conducted on the summer range of the Kangerlussuaq-Sisimiut caribou herd in West-Greenland, located approximately 20 km west of Kangerlussuaq (67°6.8'N, 50°20'W, 50–500 m.a.s.l). The area is occupied by caribou during and following parturition from early May until late June [23,46,47] and by a residential muskox population [48]. The study site is located on non-carbonate mountain bedrock and the dominant plant community type is defined as low shrub tundra [49]. Mean annual precipitation in the study area is relatively low during the greater part of the growth season. From May through August, mean monthly precipitation is 8, 15, 24 and 33 mm, respectively (Danish Meteorological Institute; ). Mean monthly temperatures for the same period are 2.5°, 8.6°, 10.7° and 8.2°C, respectively. The north-facing and cooler slopes are dominated by Labrador tea (*Ledum palustre*), while xerophyllic plants (e.g. *Kobresia myosuroides *and *Carex supine*) characterize the warmer, more south-facing slopes, with dense patches of dwarf birch (*Betula nana*) interspersed with grayleaf willow (*Salix glauca*) at lower elevations. The moister valley floors are typically dominated by greens of *Poa pratensis *[47].

### Experimental design

The warming treatment was conducted inside and outside of two, 800 m^2^, circular caribou- and muskox-proof exclosures that were erected in early June of 2002. The 1.5 m tall exclosures were constructed of woven wire and steel fence posts. Two comparable areas of the same size, elevation and vegetation type were established as controls at the same time the exclosures were erected. In spring 2003, we initiated the warming experiment. Circular open-top chambers (OTCs) with a basal diameter of 150 cm, a side angle of 60°, and a height of 40 cm were constructed according to the protocol for the International Tundra Experiment (ITEX) program. On each of the exclosed and control sites, six experimental plots were randomly selected for a total of 24 plots; OTCs were randomly assigned to three of the plots within each area. This resulted in a nested design in which each warming plot was nested within the corresponding exclosure or control area. There were four different treatment combinations: ungrazed and warmed (UW), ungrazed and ambient (UA), grazed and warmed (GW), and grazed and ambient (GA).

The following rejection criteria were used for plot selection: all functional plant groups of interest had to be represented in each plot (shrubs, graminoids, and forbs, but also lichens and mosses); all shrubs had to be small enough to entirely grow within the plot; and plots had to be at least 2 m away from the fence. The warming manipulation lasted for 22 days in June 2003 (from June 4 until June 25), before the OTCs were removed from all plots, both inside and outside the exclosures, allowing ungulate herbivores access to the un-exclosed plots. This was practiced for all the seasons to give the plots exposed to grazing and those excluded from grazing the same warming treatments. During the 2004 growing season, the warming treatment lasted from May 19 until July 11, in 2005 warming lasted from May 23 until July 17, and in 2006 it lasted from May 22 until July 29.

To be able to investigate the influence of both grazing and warming on aboveground biomass production, we needed to find a balance between optimized warming and lack of interference with herbivory. Because the warming treatment might be more important in spring and early summer, the OTCs were in place from the beginning of the growing season towards the peak of the growing season. For all years, except for 2003, the OTC's were in place while there was still snow cover on the ground. We recognize that our warming treatment might not have advanced spring warming as fully as possible. However, the need to minimize interference with herbivory required that the OTCs were removed annually. By removing the OTCs from the peak of the growing season until the end of winter, we tried to limit the lack of warming treatment as much as possible while allowing sufficient opportunity for herbivory.

### Sampling

To assess the effects of the OTC treatment on microclimate, we monitored soil and near-surface temperature in control and warmed plots from mid June until mid July each year. From mid July, we left the study area to minimize any effects of our presence on animal grazing behavior, and for that reason was not able to extend the temperature monitoring. Digital thermometers with the sensor placed approximately 5 cm above the ground in the vegetation logged max/min data for 6 consecutive days each year to monitor near-surface temperature inside and outside of the OTCs (Taylor Precision Products; ). Soil temperatures were measures with a probe inserted 10 cm into the soil. We ensured that the sensors were not directly exposed to sunlight.

Plant sampling was conducted using a non-destructive point-intercept method [50,51], because this study is part of a long-term experiment. An aluminum quadratic frame, 0.25 m^2^, with adjustable steel legs and a 0.25 m^2 ^plexi-glass plate with 20 randomly drilled holes on top was secured over each plot during sampling. A metal pin, 3 mm in diameter, was vertically lowered through each of the holes until it hit the ground or cryptogam layer. Dead plant tissue attached or on the ground was recorded as litter. Every encounter between the pin and the vegetation was recorded according to different functional groups (shrubs, forbs, graminoids etc.). *B. nana *was the dominant species in the plots, and was the only shrub species present in all plots. In this investigation, *B. nana *is the only shrub species considered. Typical forb species found in the study area included *Cerastium alpinum*, *Draba nivalis*, *Polygonum viviparum*, *Stellaria *sp., *Melandrium *sp. As for graminoids the species found within this functional group consisted of species from the genera *Poa*, *Festuca, Carex*, *Kobresia *and *Luzula*. The number of intercepts gives an index of the biomass of each plant functional group in each plot. Each plot was permanently marked for repeated sampling. Measurements were conducted at the initiation and end of the warming treatment (usually at the peak of the growing season) as well as at the end of the growing season in August.

### Biomass estimation

To convert the intercept frequency to biomass values for the permanent vegetation plots, 20 additional plots were chosen during the peak of the growing season in 2003, with emphasis on representing as many of the functional groups and as wide a range of biomass as possible. The plots were chosen from the same area and vegetation types as the permanent plots. After the intercept frequency was recorded, vegetation in each of the plots was clipped to ground level, sorted into functional groups, and dried for 24 hours at 60°C before being weighed [50,51]. For shrubs, leaf and stem biomass were parameterized separately. The point frequencies were then regressed against the biomass for each functional group, with biomass as the dependent variable and intercept frequency as independent variable. We compared an untransformed linear model, an exponential model with the dependent variable LN-transformed, and a multiplicative model with both the dependent and independent variable LN-transformed, all of which had been previously considered [50]. The best regression model was chosen based on the highest value for the coefficient of determination (r^2^). The multiplicative model (y = ax^b^) gave the best fit for graminoids and forbs (Graminoids, r^2 ^= 0.65, F = 33.79, p < 0.001; Forbs, r^2 ^= 0.51, F = 15.39, p = 0.001), while the untransformed linear model (y = bx + a) gave the best fit for *B. nana *leaves and stem (*B. nana *leaves, r^2 ^= 0.64, F = 32.43, p < 0.001; *B. nana *stem, r^2 ^= 0.50, F = 17.92, p < 0.001). The resulting equations used to calculate biomass from intercepts were as follows: graminoids, y = 0.125x^0.872^; forbs, y = 0.478x^0.491^, *B. nana *leaves, y = 1.09x + 4.76 and *B. nana *stem, y = 8.30x + 30.14. All the regression-based translations of the pin-intercepts are presented as g/m^2^. The results are presented as biomass and not pin intercepts because biomass is a more biologically relevant way of analyzing and presenting the data in this study. However, the intercepts were also analyzed, and provided the same results as for analyses of biomass.

### Vertebrate herbivore counts

On a daily basis we conducted counts of caribou and muskoxen that were observed in each control site or areas most adjacent to the control site (Table 4). These numbers were entered as predictor variables in our statistical analyses, but were not significant, and subsequently omitted from the analyses.

**Table 4:** Daily mean numbers of caribou and muskoxen observed in each control site each year from 2003 until 2006, Kangerlussuaq, Greenland.

Year	Control site	Daily mean # caribou	Daily mean # muskoxen	Daily mean # caribou + muskoxen
2003	Site 1	0.27	0.40	1.53
	Site 2	1.40	0.07	1.47
2004	Site 1	0.17	0.83	1.00
	Site 2	0.50	0.72	1.22
2005	Site 1	1.11	0.05	1.16
	Site 2	16.4	0.37	16.74
2006	Site 1	1.88	1.12	3.00
	Site 2	0.82	0.88	1.71

### Analyses

There was an outbreak of caterpillars of the moth *Eurois occulta *during the 2004 and 2005 growing seasons that altered the aboveground biomass of the functional groups analyzed in this paper. Although the caterpillars made a strong impact on the plants, the effect on aboveground biomass should be equal among the treatment and control plots, and hence should not influence the between-treatment comparisons presented here. The effect of the moth outbreak has been the focus of a separate paper [40] and is not analyzed in detail here. Analysis in the other paper shows that the caterpillar density did not interact with the warming or exclosure manipulations on aboveground biomass. There were consistent effects of reduction in aboveground biomass across treatments [40]. The results presented here are from 2003, 2004, 2005 and 2006, with data collected during the peak of the growing season in July, except data from 2003 that were collected at the end of June. This was done to minimize any effects that date of sampling would have on the between years comparisons.

Main effects of warming on mean near-surface and soil temperatures were evaluated by an ANOVA model with plot and Julian date as random factors to control for any between plot variation and time of measurement effects. The daily mean temperatures were calculated as the average between the maximum and minimum temperatures recorded in each plot in a 24 hour period. Due to the nested design of this experiment, a nested-ANOVA was used to analyze the effects of the warming and exclosure treatments on aboveground biomass of the main plant functional groups. All data were analyzed at the plot level. A site term was included as a random factor to control for any site variation, while year was included as a random factor to control for any interannual variation besides those due to the experimental treatments.

To quantify changes in aboveground biomass, we distinguished between "primary", "secondary" and "cumulative" responses. The primary response was the change observed between 2003 and 2004, while the secondary response was between 2005 and 2006 and the cumulative response was the observed change between the initiation of the warming experiment in 2003 and 2006. The reason for focusing on these periods was to avoid most of the effects of the *E. occulta *outbreak that was initiated in 2004 and peaked in 2005, before returning to unnoticeable numbers in 2006. All statistical tests were performed using the statistical program SPSS version 10.0 for Windows.

## Authors' contributions

EP conceived of the study, CP & EP set up the experiment and conducted the fieldwork, CP performed the analyses and writing, and EP assisted with the analyses and writing.
